# Cytogenetic markers applied to cytotaxonomy in two soybean pests: *Anticarsia gemmatalis* (Hübner, 1818) and *Chrysodeixis includens* (Walker, 1858)

**DOI:** 10.1371/journal.pone.0230244

**Published:** 2020-03-11

**Authors:** Brenda Rafaella Da Silva Magalhães, Daniel Ricardo Sosa-Goméz, Jaqueline Fernanda Dionísio, Felipe Cordeiro Dias, Joana Neres Da Cruz Baldissera, Matheus Pires Rincão, Renata Da Rosa

**Affiliations:** 1 Laboratório de Citogenética Animal, Departamento de Biologia Geral, Universidade Estadual de Londrina, Brasil; 2 Laboratório de Biologia Molecular de Artrópodes e Entomopatógenos, Embrapa Soja, Londrina, Brasil; Western Kentucky University, UNITED STATES

## Abstract

*Anticarsia gemmatalis* (Hübner, 1818) and *Chrysodeixis includens* (Walker, 1858) are species of Lepidoptera that cause great damages in the soybean plantations of Brazil. Despite the importance they have in this regard, there are no studies on the chromosomal organization of these species and recently, *A*. *gemmatalis*, which belonged to the Noctuidae family, was allocated to the Erebidae family. Therefore, the objective of this paper was to analyze, through conventional and molecular cytogenetic markers, both species of Lepidoptera. A 2*n* = 62 was observed, with ZZ/ZW sex chromosome system and holokinetic chromosomes for both species. There was homogeneity in the number of 18S rDNA sites for both species. However, variations in heterochromatin distribution were observed between both species. The cytogenetic analyses enabled separation of the species, corroborating the transference of *A*. *gemmatalis*, from the family Noctuidae to the family Erebidae, suggesting new cytotaxonomic characteristics.

## Introduction

The order Lepidoptera (Linnaeus, 1758) is composed of approximately 15,000 genera and 157,000 species [1]. The representatives of this order have wings covered by scales, and are popularly known as butterflies and moths, and include many species of great economic importance. They are holometabolous insects [2] and primarily phytophagous, when in larval stages making many of these insects pests. Among the Lepidoptera, *Anticarsia gemmatalis* (Hübner, 1818) and *Chrysodeixis includens* (Walker, 1858) stand out as soybean crop pests in Brazil.

Approximately 6,000 lepidopteran species have economic importance, and it is estimated that 25% are classified in the superfamily Noctuoidea. Morphological classifications performed by Kitching [3] that were associated with molecular data (mitochondrial and nuclear gene sequences) obtained in the study by Zahiri et al. [4], separated the super family into six clades: Oenosandridae; Notodontidae; Erebidae; Euteliidae; Nolidae; and Noctuidae. Wing venation is a characteristic that classifies the superfamily into four sections, one of which groups both the Erebidae family and the Noctuidae family [5], which makes it difficult to assign species to the correct family.

*A*. *gemmatalis* and *C*. *includens* were previously classified as part of the Noctuidae family, until studies by Zahiri et al. [5] confirmed that *A*. *gemmatalis* possesses greater similarity (BS ≥ 86) to the subfamily Eulepidotinae of the Erebidae family than the subfamily Catocalinae of the Noctuidae family, and transfered it to the Erebidae. Meanwhile, *C*. *includens* continues to belong to the subfamily Plusiinae of the Noctuidae family [6]. The definitions of the Noctuoidea classifications are not yet well elucidated [4,5,7–9]. In this way, cytogenetics is an important tool to aid in the decision-making process of changes in the systematic classification of different organisms, as well as in the development of cytotaxonomic studies involving evolution.

There are several conventional cytogenetic studies in Lepidoptera that have so far demonstrated some common characteristics such as holokinetic chromosomes and sex chromosomal systems [10–12]. Females, the heterogametic sex, are associated with achiasmatic meiosis during prophase I of oocyte meiosis, giving the species a different meiotic behavior between autosomes and sex chromosomes [13, 14].

The most common diploid number in the order Lepidoptera is 2*n* = 62 (30A + ZW) [15–17]; however, numbers of n = 10 to 108 are described, such as those of the genus *Polyommatus* [18]. Conventional cytogenetic (C, G, Q, R, and NOR) techniques are poorly performed in order because they chromosomes are small, numerous, and uniform [15, 19–23], and also due to the difficulty of obtaining the chromosomes.

Nguyen et al. [17] carried out a study on the localization of 18S ribosomal DNA clusters (18S DNAr) in 30 species of five super families (Tortricoidea, Pyraloidea, Bombycoidea, Papilionoidea, and Noctuoidea) and used the location of this sequence as a marker for evolutionary studies. These studies revealed that 18S rDNA has a preferential distribution in the interstitial region in the Noctuoidea superfamily, which includes the families Noctuidae and Erebidae.

Biological, genetic, and cytogenetic knowledge of Lepidoptera contributes to the achievement of new strategies for the control of these pests [6, 23–25]. Although there are cytogenetic studies in Lepidoptera, there are no reports on the karyotypic structure of *A*. *gemmatalis* and *C*. *includens*. In addition, studies related to the population structure of these soybean pest insects are scarce. Thus, the objective of this work was to analyze the classical and molecular cytogenetics of these two species to elucidate the evolutionary relationships between them, as well as to evaluate, through the chromosomal analysis, the new taxonomic classification of these species.

## Materials and methods

### Biological material and chromosome preparations

Specimens of *A*. *gemmatalis* (30 samples) and *C*. *includens* (20 samples) were collected and maintained by the Laboratory of Molecular Biology of Arthropods and Entomopathogens (Embrapa Soja, CNPSO–Brazil). After application of 0.2% colchicine intraperitoneally for 6 hours, the testes and ovaries were dissected, hypotonized in distilled water for 10 min, fixed in methanol and acetic acid solution (3:1, v:v), and stored in a freezer −20 °C. For the preparation of the slides, the material was submerged in 60% acetic acid for approximately 15 min and subjected to the SteamDrop technique [26]. The slides were dried and stained with Giemsa 2% for conventional analyses.

### C-banding and fluorochromes

The constitutive heterochromatin was identified by the C-band [27]. After the C-banding, the slides were stained with Giemsa 2% and fluorochromes Cromomycin A_3_ (CMA_3_) and 4′-6-diamidino-2-phenylindole (DAPI) for the detection of the GC and AT-rich chromosomal regions, respectively [28].

### Isolation of 18S rDNA and fluorescence *in situ* hybridization

Total DNA was extracted from the muscle tissues of *A*. *gemmatalis* samples following the protocol, with modifications [29] and purified with 7.5 M ammonium acetate and 70% and 100% ethanol. The concentration and purity of the DNA was determined in NanoDrop™ and the samples were diluted to concentrations of 100 ng/μl for the procedures that follow.

18S rDNA was amplified with the primers 18S-Gal *Forward* 5′-CGATACCGCGAATGGCTCAATA-3′ and 18S-Gal *Reverse* 5′-ACAAAGGGCAGGGACGTAATCAAC-3′ [30]. Polymerase chain reaction (PCR) was performed for a final volume of 25 μl containing 12.5 μl of GoTaq^®^ Green Master Mix 2X (Promega), 1 μl of DNA (100 ng/μl), 0.5 μl of each primer (10 mM), and 10.5 μl H_2_O. Amplification was confirmed on 1% agarose gel (with SYBR Safe^™^ dye, Invitrogen) and purified with 7.5M ammonium acetate and 70% and 100% ethanol.

PCR products obtained from 18S rDNA were sequenced automatically (ABI 3500 XL Applied Biosystems). Clearance of DNA sequences, sequence quality analysis, and assembling were performed in the Mega 7.0 software [31] and BioEdit v.7.2.6.1 [32]. The consensus sequences were compared to other sequences previously deposited in the National Biotechnology Information Center (NCBI) Database using the BLAST Search tool. (http://www.ncbi.nlm.nih.gov/blast).

Fluorescence *in situ* hybridization (FISH) was performed according to Pinkel et al. [33]. The amplified product of 18S rDNA was labeled with Biotin-11-dUTP by PCR. The probes were detected with Avidin-FITC and contrasted with DAPI and propidium iodide. The slides submitted to the different cytogenetic techniques were analyzed in a Leica DM 2000 fluorescence photomicroscope equipped with a DFC 300 FX camera with Motic Images Plus 3.2 image analysis software.

### Cytochrome C Oxidase subunit I (COI) gene analysis

To confirm the species and their taxonomic classification, the COI gene of the two species was amplified according Folmer et al. [34] the primers selected were LCO1490 5'-GGTCAACAAATCATAAAGATATTGG-3' and HCO2198 5'-TAAACTTCAGGGTGACCAAAAAATCA-3'. PCR was performed for a 25μl final volume containing 12.5μl GoTaq^®^ Green Master Mix 2X (Promega), 1μl DNA (100ng / μl), 0.5μl of each primer (10mM) and 10.5μl of water. After amplification, confirmed on 1% agarose gel, the products were purified with 7.5M ammonium acetate and 70 and 100% ethanol. PCR products were sequenced in an automated sequencer (ABI 3500 XL Applied Biosystems). DNA sequence cleaning, sequence quality analysis and contig assembly were performed using the Mega 7.0 software [31] and BioEdit v.7.2.6.1 [32]. Consensus sequences were compared to other sequences previously deposited in the National Biotechnology Information Center (NCBI) Database, with the aid of the BLAST Search tool. (http://www.ncbi.nlm.nih.gov/blast).

## Results and discussion

**Fig 1:**
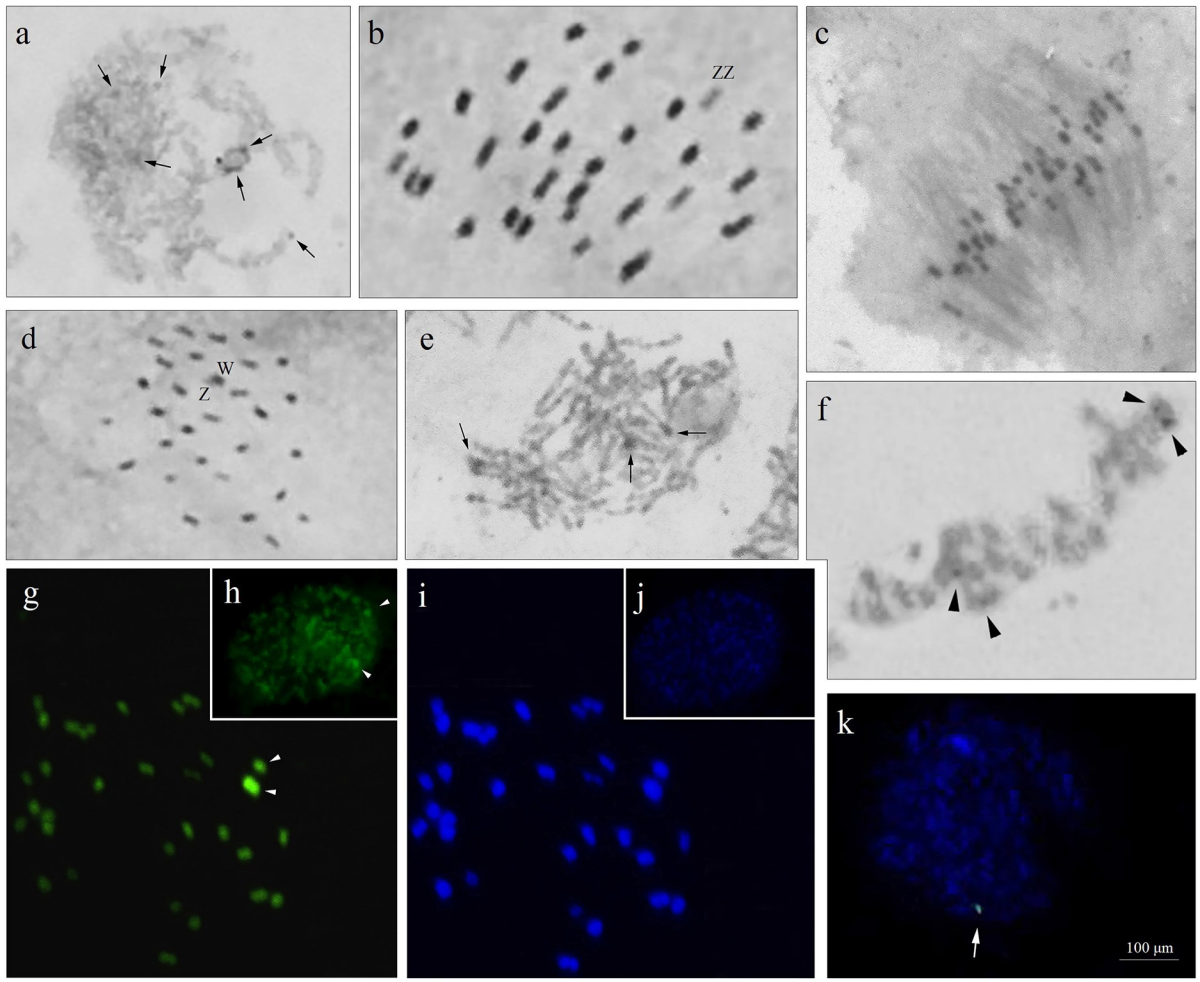
Meiocytes of *A*. *gemmatalis* submitted to conventional staining by Giemsa (a-f) and fluorochromes CMA_3_/DAPI (g-j). (a) pachytene; (b) male metaphase II; (c) anaphase II; (d) female metaphase II; (e) pachytene submitted to the C-banding; (f) male metaphase II with C-banding. arrows indicate the heterochromatic regions; (g) metaphase I. Note the two divalent CMA_3_^+^; (h) interphase nucleus; (i) metaphase I (DAPI); (j) interphase nucleus; (k) interphase nucleus after fluorescence *in situ* hybridization with biotin-labeled 18S DNAr probe and counterstained with DAPI. Note the detail of the unique marking with 18S rDNA.

**Fig 2:**
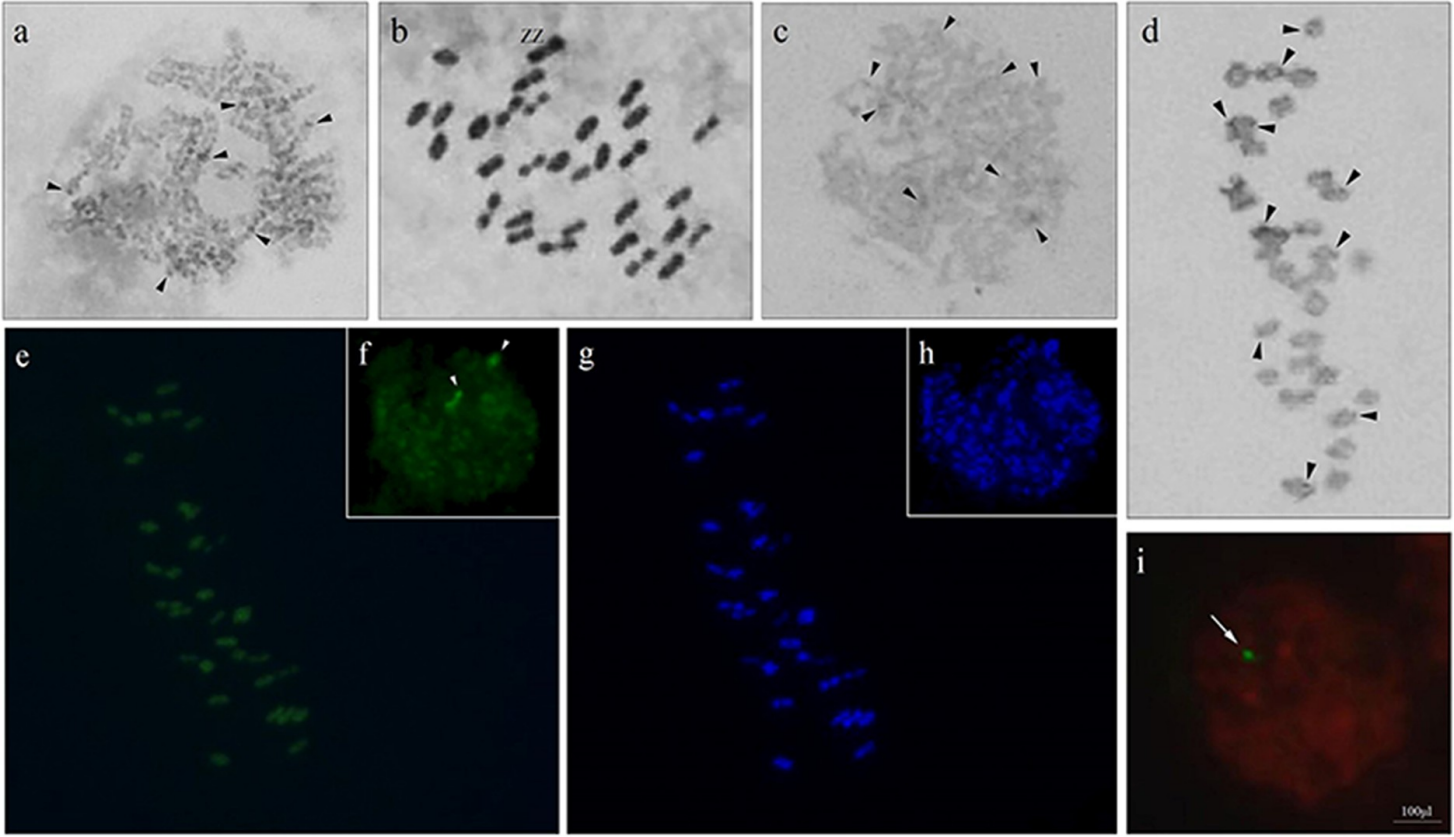
Meiotic cells of *C*. *includens* in different staining by Giemsa (a-d) fluorochromes CMA_3_/DAPI (e-h). (a) pachytene; (b) metaphase II; (c) pachytene submitted to the C-banding; (d) metaphase II with C-banding; (e) metaphase II, arrows indicate GC-rich sites; (f) interphase nucleus, the arrowheads indicate the CMA_3_^+^ marks; (g) metaphase II, observe the greatest number of DAPI^+^ marks; (h) interphase nucleus stained with DAPI, observe the AT-rich marks; (i) interphase nucleus after fluorescence *in situ* hybridization with 18S DNAr probe.

*A*. *gemmatalis* (Erebidae) and *C*. *includens* (Noctuidae) have many cytogenetic features in common. Cytogenetic analyses by conventional staining allowed the identification of the diploid number as well as the behavior of the chromosomes during the cell division. The karyotype formed by 2*n* = 62 (30A + ZZ/ZW) with holocentric chromosomes of similar sizes and formats (Figs 1 and 2), allowed for observation that both species have a karyotype similar to that of the other species of the order Lepidoptera already studied, following the modal number *n* = 31, which shows a large conservation in the karyotype macrostructure for Lepidoptera [15–17, 35].

In pachytene, for both species, the formation of bivalents was observed and chromosomes were negative heteropycnotic (Figs 1a and 2a); however, in *C*. *includens* there was a larger number of these regions. As previously described, lepidopteran chromosomes have uniform sizes and formats, which often hampers studies that require the visualization of bands obtained by chemical and enzymatic treatments, requiring pretreatments that help to obtain satisfactory phases for the analyses [15, 19, 22, 23].

In metaphase II, chromosomes were aligned on the equatorial plate, including possible sex chromosomes. In *C*. *includens*, these chromosomes presented positive heteropycnosis (ZZ) (Fig 2b) whereas in *A*. *gemmatalis* ZZ chromosomes were negative heteropycnotics (Fig 1b) and the W chromosome exhibited positive heteropycnosis (Fig 1d) in female metaphase II. The pairing of chromosomes with heteropycnosis and heteromorphism suggests that the possible sex chromosomes present the touch-and-go conformation and occurrence of inverted meiosis.

The sex chromosome system found most frequently among Lepidoptera is the ZZ/ZW, in which the female has the heterogametic chromosomal pair. Sex chromosome systems ZZ/Z0, ZZ/ZW_1_W_2_, and Z_1_Z_1_Z_2_Z_2_/Z_1_Z_2_W [13] have also been described. In the present paper, the identification of a pseudobivalent with heteromorphism and heteropycnosis in *A*. *gemmatalis* led to the association of an achiasmatic meiosis of these sex chromosomes in females, identifying them as ZW (Fig 1d), as well as already described for *Bombyx mori*, *Ephestia kuehniella*, and *Leptidea amurensis* [13, 14]. It was not possible to make inference about such data in females of *C*. *includen*s; however, in metaphase II (Fig 2b) of males, a larger sized pair of chromosomes was observed, which we can suggest as being the pair of sex chromosomes.

The chromosomes were holocentric to both species, as reported previously in other Lepidoptera [17, 18]. Anaphase II (Fig 1c) confirmed the differentiated behavior of the holocentric chromosomes and it is possible to visualize the positioning of the spindle fibers with the kinetochore along the chromosomes. In addition, it is possible to observe the early migration of some chromosomes, which may correspond to the sex chromosomes.

Although conventional analysis revealed karyotypic conservation, banding techniques differentiated the two species. In pachytene, more condensed regions were observed in the two species, stained darker (heterochromatic) along the chromatids and interspersed with lighter or eucromatic regions (Figs 1e and 2c). In *A*. *gemmatalis*, a smaller amount of heterochromatin was observed, with two bivalents labeled (Fig 1f). These bands are difficult to characterize, and their identification depends on the degree of condensation of the chromosomes in other meiotic phases, as in pachytene. In *C*. *includens*, several bivalents presented two labels (Fig 2d), which were observed in greater quantities and intensity than in *A*. *gemmatalis*. Goodpasture [20] applied the C-banding technique in pachytene of two species of Lepidoptera, *Pyrgus oileus* (Hesperiidae) and *Prodenia ornithogalli* (Noctuidae), and viewed interstitial markings. In interphase nuclei, the nucleolus was evidenced and associated with the chromosomes with the terminal markings. In the present study, this type of association can be confirmed in the pachytene of *A*. *gemmatalis*.

These differences were also observed in fluorochrome staining. DAPI staining discriminated slight markings on *C*. *includens*, while in *A*. *gemmatalis* no DAPI^+^ regions were observed. For *A*. *gemmatalis*, CMA_3_ staining revealed brighter markings on two bivalents (Fig 1g) and two nuclear markers (Fig 1h), suggesting that they are the same as those identified by the C-banding. Some discrete marks could be observed in other bivalents (Fig 1g). With DAPI staining, it was not possible to verify any marking, either in metaphase I or in interphase nucleus (Fig 1i and 1j). In *C*. *includens*, it is also possible to identify bivalents in metaphase II (Fig 2e) and two nuclear markers with CMA_3_ (Fig 2f), besides some discrete markings on several chromosomes. The DAPI staining identified discrete DAPI^+^ bands in metaphase II (Fig 2g), but no positive marking that allowed visualization in the nucleus (Fig 2h).

Our results have suggested that these chromosomes are the same identified by the C-banding, inferring that the chromosomes present regions rich in C/G. We can also suggest that these chromosomes are related to the sex chromosomes or to the sites of rDNA 18S, as identified in *Mamestra brassicae* [21, 36].

This differentiation of heterochromatic blocks detected both by the C-band technique and by the coloration of base-specific fluorochromes may result from heterochromatinization processes or the presence of transposable elements. These events have previously been suggested in studies where there were differences in the presence of intra- and inter-specific heterochromatic blocks in grasshopper populations [37, 38] and in beetles [39]. In addition to protecting the genome from potentially mutagenic events, heterochromatin formation suggests that they are crucial sequences for the functional organization of important chromosomal structures, such as telomeres and centromeres [40], relating it to the karyotype of evolutionary studies.

The partial consensus sequence of *A*. *gemmatalis* 18S rDNA showed a length of 1034 bp and 94%–96% similarity to the conserved 18S rDNA region of several other lepidopteran species (accession number MN989998). The sequence obtained by sequencing was compared with other sequences and the results obtained were similar to other Lepidoptera, such as *Hyles lineata* (accession number: AF423786.1), *Antheraea assama* (accession number: KY676860.1), *Neutral tanadema* (accession number: KR068959.1), *Helicoverpa armigera* (accession number: KT343378.1), and *Plutella xylostella* (accession number: JX390653.1), among other species deposited in GenBank (NCBI). The 18S ribosomal DNA sequence for *C*. *includens* showed a 920bp fragment (accession number MN990034) with a maximum identity of 98% with other lepidopterans, the most similar being *H*. *armigera* (accession number: KT343381.1). By the FISH technique, it was possible to identify only one block of 18S rDNA in the interphase nuclei of *A*. *gemmatalis* and in pachytene of *C*. *includens* (Figs 1k and 2i).

The identification of clusters of 18S rDNA by FISH in Lepidoptera is very variable, directly related to the fissions, fusions, and translocations that occur and are facilitated by the characteristics of holocentric chromosomes [18]. Thus, when this event occurs, as in *Leptidea amurensis*, it is possible to carry out a phylogenetic study and to identify the karyotype evolution of the species by taking into account only the cluster of rDNA 18S [14].

The pattern of RONs in the Noctuoidea superfamily is the presence of only an interstitial marking, except for *Spodoptera latifascia* and *S*. *descoinsi*, which present more than one marking [17, 41]. A labeling in the nuclei of *A*. *gemmatalis* and *C*. *includens* confirms that labeling which has been observed in the superfamily that corroborates the karyotypic conservation regarding the number of this sequence; however, due to the difficulty of observing these chromosomes, it was not possible to observe the location of the 18S rDNA in metaphase chromosomes, which made it impossible to determine the position of this site.

The Insect COI (LCO1490 and HCO2198) primers of the Cytochrome C Oxidase subunit I (COI) gene amplified 750 bp to 800 bp mtDNA fragments in both species (Genbank accession number MN869915; BOLD accession number AAA6794), confirmed by 1% agarose gel electrophoresis. BLAST Graphics and Taxonomy results supported the placement of *A*. *gemmatalis* in Erebidae and *C*. *includens* in Noctuidae by the comparison with other sequences of the COI gene from Lepidoptera (S1 Fig).

Although we observed a great karyotypic conservation in diploid number, chromosome size, sexual systems, and number of 18S rDNA sites, differences in heterochromatin distribution patterns were observed. These procedures are relevant in the search for species-specific and population-specific chromosome markers in the contribution of evolutionary analyses and deserve attention, since these are the first results of the species studied obtained. Thus, our data support the proposed by Zahiri et al. [4], confirming the chromosome differentiation between the species studied and the phylogenetic position of the genus *Anticarsia* in Erebidae. Thus, our cytogenetic data corroborate the new position of the species, and bring to light the differences between the representative species of the orders Erebidae and Noctuidae.

## Supporting information

S1 Fig BLAST Graphics and Taxonomy results for the Cytochrome Oxidase SubUnit I (COI) gene fragment. C. includens (a) and A. gemmatalis (b).
(PDF)
