# Supplementary material for: Cytogenetic markers applied to cytotaxonomy in two soybean pests: Anticarsia gemmatalis (Hübner, 1818) and Chrysodeixis includens (Walker, 1858)
Source: PLoS One. 2020 Mar 11;15(3):e0230244. doi: 10.1371/journal.pone.0230244 (PMC7065768; doi:10.1371/journal.pone.0230244)
Supplement: S1 Fig — C. includens (a) and A. gemmatalis (b). (PDF) [file pone.0230244.s001.pdf]

S1_Fig. BLAST Graphics and Taxonomy results for the Cytochrome Oxidase SubUnit I (COI) gene fragment. *C. includens* (a) and *A. gemmatalis* (b)

(a)

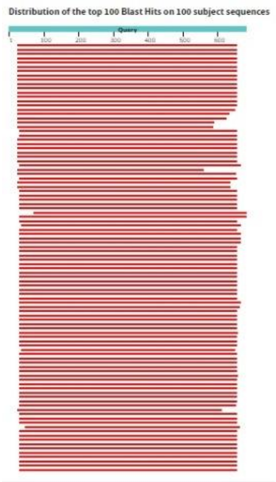

Taxonomy	Number of hits	Number of Organisms	Description
⊟Lepidoptera	236	41	
. ⊟Obtectomera	221	37	
. . ⊟Noctuoidea	220	36	
. . . ⊟Noctuidae	219	35	
. . . . ⊟Plusiinae	207	23	
. . . . . ⊟Chrysodeixis	136	8	
. . . . . . Chrysodeixis includens	81	1	Chrysodeixis includens hits
. . . . . . ⊟unclassified Chrysodeixis	2	2	
. . . . Chrysodeixis sp. sc_01548	1	1	Chrysodeixis sp. sc_01548 hits
. . . . Chrysodeixis sp. sc_09829	1	1	Chrysodeixis sp. sc_09829 hits
. . . . . . Chrysodeixis illuminata	2	1	Chrysodeixis illuminata hits
. . . . . . Chrysodeixis acuta	19	1	Chrysodeixis acuta hits
. . . . . . Chrysodeixis chalcites	14	1	Chrysodeixis chalcites hits
. . . . . . Chrysodeixis argentifera	1	1	Chrysodeixis argentifera hits
. . . . . . Chrysodeixis eriosoma	17	1	Chrysodeixis eriosoma hits

(b)

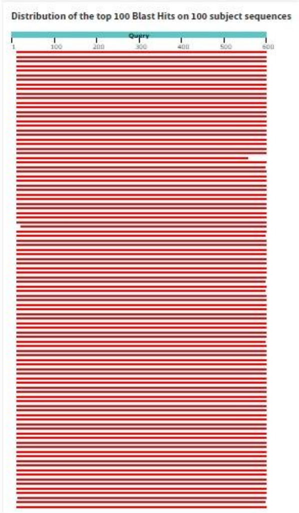

Organism	Blast Name	Score	Number of Hits	
Obtectomera	moths		105	
. Noctuoidea	moths		94	
. . Erebidae	moths			
. . . Anticarsia gemmatalis	moths	1088	45	Anticarsia gemmatalis hits
. . . Anterastria atrata	moths	878	2	Anterastria atrata hits
